# Extracts from *Sageretia thea* reduce cell viability through inducing cyclin D1 proteasomal degradation and HO-1 expression in human colorectal cancer cells

**DOI:** 10.1186/s12906-019-2453-4

**Published:** 2019-02-08

**Authors:** Ha Na Kim, Gwang Hun Park, Su Bin Park, Jeong Dong Kim, Hyun Ji Eo, Ho-Jun Son, Jeong Ho Song, Jin Boo Jeong

**Affiliations:** 1 Department of Medicinal Plant Resources, Andong National University, 1375 Gyeongdong-ro, Andong, Gyeongsangbuk-do 36729 Republic of Korea; 2 Forest Medicinal Resources Research Center, National Institute of Forest Science, Yongju, 36040 Republic of Korea; 3 Agricultural Science and Technology Research Institute, Andong National University, Andong, 36729 Republic of Korea

**Keywords:** Anticancer, Cell viability, Cyclin D1, Heme oxygenase-1, Human colorectal cancer, *Sageretia thea*

## Abstract

**Background:**

*Sageretia thea* (*S. thea*) has been used as the medicinal plant for treating hepatitis and fevers in Korea and China*.* Recently, anticancer activity of *S. thea* has been reported, but the potential mechanism for the anti-cancer property of *S. thea* is still insufficient. Thus, we evaluated whether extracts from the leaves (STL) and branches (STB) of *S. thea* exert anticancer activity and elucidated its potential mechanism in SW480 cells.

**Methods:**

MTT assay was performed for measuring cell viability. Western blot and RT-PCR were used for analyzing the level of protein and mRNA, respectively.

**Results:**

Treatment of STL or STB decreased the cell viability and induced apoptosis in SW480 cells. Decreased level of cyclin D1 protein was observed in SW480 cells treated with STL or STB, but no change in cyclin D1 mRNA level was observed with the treatment of STL or STB. MG132 blocked downregulation of cyclin D1 protein by STL or STB. Thr286 phosphorylation of cyclin D1 by STL or STB occurred faster than downregulation of cyclin D1 protein in SW480 cells. When SW480 cells were transfected with T286A-cyclin D1, cyclin D1 degradation by STL or STB did not occur. Inhibition of GSK3β and cyclin D1 nuclear export attenuated STL or STB-mediated cyclin D1 degradation. In addition, STL or STB increased HO-1 expression, and the inhibition of HO-1 attenuated the induction of apoptosis by STL or STB. HO-1 expression by STL or STB resulted from Nrf2 activation through ROS-dependent p38 activation.

**Conclusions:**

These results indicate that STL or STB may induce GSK3β-dependent cyclin D1 degradation, and increase HO-1 expression through activating Nrf2 via ROS-dependent p38 activation, which resulted in the decrease of the viability in SW480 cells. These findings suggest that STL or STB may have great potential for the development of anti-cancer drug.

## Background

Globally, cancer is considered as one of major health problems [1]. Among cancers, colorectal cancer is fourth most common malignant tumor, the third leading cause of cancer deaths worldwide [2]. Although many anticancer drugs have been developed for clinical use for the treatment of colorectal cancer, the long-term use of these anticancer drugs causes many side effects [3]. For example, fluorouracil causes neutropenia, stomatitis and diarrhea, and irinotecan induces bone marrow suppression, nausea and alopecia [3]. In addition, oxaliplatin has been reported to cause dysesthesias and renal dysfunction [3], Thus, medicinal plant resources are being utilized for complementary alternative treatment of cancer [4], and have been regarded as the promising sources for developing anticancer drugs [5]. Indeed, clinically used anticancer drugs such as taxol, vinblastine, vincristine, irinotecan and camptothecin have been developed using plant-derived products [6].

In cell cycle progression, cyclin D1 complexed with CDK4 and 6 induces phosphorylation of retinoblastoma protein which promotes the progression of cell cycle [7]. In addition, cyclin D1 is involved in the transcriptional activation of genes that induce cell growth, genetic instability, invasion, and metastasis [7–10]. In addition to the cell cycle control function, cyclin D1 has also been reported to modulate apoptosis [11]. Inhibition of cyclin D1 expression induced apoptosis of various cancer cells [12, 13]. On the other hand, upregulation of cyclin D1 inhibited apoptosis in human choriocarcinoma cells [14]. Cyclin D1 overexpression has been observed in 63.6% of colorectal cancer [15] and cyclin D1 has been known to be participated in the growth and differentiation of colorectal cancer [16]. Thus, cyclin D1 has been regarded as a hallmark of colorectal cancer and a potential target of colorectal cancer treatment.

Recently, HO-1 has been proposed as a potential molecular anticancer target [17]. HO-1, known to as heat shock protein 32 catalyzed heme degradation into bilirubin, iron ion and carbon monoxide. HO-1 activation inhibits oxidative cell damage induced by hydrogen peroxide and UV, production of inflammatory mediators, and induction of high-glucose [18–21]. However, HO-1 has been reported to have a multifaceted role in cancer development [17]. HO-1 increases the growth of several cancer cells such as pancreatic cancer, melanoma and rhabdomyosarcoma [22–24]. In the opposite effect of HO-1, the overexpression of HO-1 exerts anti-proliferative activity in breast cancer, prostate cancer and colorectal cancer cells [25–27]. In addition, HO-1 suppresses cell migration and xenograft tumor growth in hepatocellular carcinoma [28].

As one of the Rhamnaceae family, *Sageretia thea* (*S. thea*) has been commonly known as Chinese sweet plum or Chinese bird plum [29]. *S. thea* as traditional herbal medicine has been treated for hepatitis and fevers in Korea and China [29, 30]. In pharmacological study, the fruits from *S. thea* have been reported to exert anti-oxidant, anti-diabetes and anti-melanogenesis activity [30, 31]. The leaves of *S. thea* inhibited the oxidation of low-density lipoprotein through its anti-oxidant activity and HIV type 1 protease [30, 32]. Recently, the leaves and branches from *S. thea* induced apoptosis in human breast cancer cells, MDA-MB-231 [33]. However, there have been no studies on the mechanisms of *S. thea* for anticancer activity. Because the elucidation of the mechanism for anticancer activity of *S. thea* is essential for the development of anticancer agent using *S. thea*, we first report the potential mechanisms of branches and leaves of *S. thea* for the anticancer activity using SW480 colorectal cancer cells.

## Methods

### Chemical reagents

LiCl (GSK3β inhibitor), MG132 (Proteasome inhibitor), PD98059 (ERK1/2 inhibitor), SB230580 (p38 inhibitor), leptomycin B (LMB, Nuclear export inhibitor), zinc protoporphyrin IX (ZnPP, HO-1 inhibitor), 3-(4,5-dimethylthizaol-2-yl)-2,5-diphenyl tetrazolium bromide (MTT), 5-Fluorouracil (5-FU) and oxaliplatin were purchased in Sigma Aldrich (St. Louis, MO, USA). Antibodies against cyclin D1, phospho-cyclin D1 (Thr286), HA-tag, p-GSK3β, total-GSK3β, p-p38, total-p38, HO-1, Nrf2, cleaved PARP, TBP and β-actin were purchased in Cell Signaling (Bervely, MA, USA).

### Preparation of the extracts of branches and leaves from *S. thea*

*S. thea* (voucher number: Jeong 201,804 (ANH)) was generously provided and formally identified by Forest Medicinal Resources Research Center, National Institute of Forest Science, Yongju, Korea. Twenty grams of the branches or leaves from *S. thea* were immersed in 500 ml of 70% ethanol and then extracted by stirring at the room temperature for 3 days. Then, the ethanol-soluble fraction was filtered, concentrated to 100 ml volume using a vacuum evaporator, and freeze-dried. The ethanol extracts from the branches (STB) or leaves (STL) of *S. thea* were stored at − 80 °C until use.

### Cell culture

SW480 cells as one of the human colorectal cancer cell lines have been widely used to investigate the potency of drugs in cancer prevention and treatment [34]. Thus, we used SW480 cells to investigate anticancer activity of STB or STL. SW480 cells obtained from Korean Cell Line Bank (Seoul, Korea) were maintained in DMEM/F-12 (Lonza, Walkersville, MD, USA) with 10% fatal bovine serum (FBS), 100 U/ml penicillin and 100 μg/ml streptomycin at 37 °C under a humidified atmosphere of 5% CO_2_. STB or STL was dissolved in dimethyl sulfoxide (DMSO). DMSO as a vehicle was used in a range not exceeding 0.1% (*v*/v).

### Cell viability

MTT assay was performed to evaluate the cell viability. SW480 cells (3 × 10^4^ cells/well) were cultured in 96-well plate for 24 h and then treated with STB or STL for the additional 24 h. After STB or STL treatment, 50 μl of MTT solution (1 mg/ml) was added to the cells and allowed to react for 2 h. After 2 h, the media was removed, and then 100 μl of DMSO was added to the cells for resulting crystals. The absorbance was measured using UV/Visible spectrophotometer (Human Cop., Xma-3000PC, Seoul, Korea) at 570 nm.

### Cell cycle analysis

SW480 cells (5 × 10^5^ cells/well) were cultured in 6-well plate 24 h. STB or STL was treated the cultured SW480 cells for 24 h. Cell cycle was analyzed with EZCell™ Cell Cycle Analysis Kit (BioVision, Milpitas, CA, USA) using a flow cytometry (FACSCalibur, USA).

### Isolation of nucleus fraction

STL or STB was treated to the SW480 cells (2 × 10^6^ cells/well) cultured in 6-well plate. In order to extract nuclear protein, the cells were washed three times with cold 1 × phosphate-buffered saline (PBS). Nuclear protein was prepared using a nuclear extract kit (Active Motif, Carlsbad, CA, USA).

### SDS-PAGE and Western blot

In order to extract the protein from the SW480 cells, the cells were washed three times with cold 1 × PBS, the cells were recovered with RIPA buffer (Boston Bio Products, Ashland, MA, USA) containing protease and phosphatase inhibitor (Sigma-Aldrich), and left at 4 °C for 30 min. After 30 min, cells were centrifuged at 15,000 × rpm at 4 °C for 10 min and the supernatant was taken for protein determination. Protein content was analyzed by BCA protein assay (Pierce, Rockford, IL, USA). The equal amount of protein was separated by SDS-polyacrylamide gel electrophoresis (SDS-PAGE) and transferred to PVDF membrane (Bio-Rad Laboratories, Inc., Hercules, CA, USA). The membrane was blocked in 5% non-fat dry milk in Tris-buffered saline containing 0.05% Tween 20 (TBS-T) for 1 h at room temperature. Each primary antibody was treated to PVDF membrane using 5% non-fat dry milk in TBS-T at 4 °C for 16 h. After washing with TBS-T, PVDV membrane was incubated with the secondary antibody for 1 h at room temperature. Then, after washing with TBS-T, the protein band was luminescent with ECL Western blotting substrate (Amersham Biosciences, Piscataway, NJ, USA) and visualized using LI-COR C-DiGit Blot Scanner (Li-COR Biosciences, Lincoln, NE, USA). The density of protein bands was calculated by UN-SCAN-IT gel version 5.1 (Silk Scientific Inc. Orem, UT, USA).

### Reverse transcriptase-polymerase chain reaction (RT-PCR)

To isolate total RNA from SW480 cells after each treatment, the cells were washed three times with cold 1 × PBS, and then total RNA was taken by a RNeasy Mini Kit (Qiagen, Valencia, CA, USA). Contents of tatal RNA were measured by UV spectrophotometer (GeneQuant 1300, GE Healthcare Life Sciences, Marlborough, MA, USA). cDNA was synthesized from 1 μg of total RNA using a Verso cDNA Kit (Thermo Scientific, Pittsburgh, PA, USA). To amplify the cDNA, PCR was performed using PCR Master Mix Kit (Promega, Madison, WI, USA). The primer sequences of cyclin D1 and GAPDH were as followed: cyclin D1: forward 5’-AACTACCTGGACCGCTTCCT-3′ and reverse 5’-CCACTTGAGCTTGTTCACCA-3′, GAPDH: forward 5’-ACCCAGAAGACTGTGGATGG-3′ and reverse 5’-TTCTAGACGGCAGGTCAGGT-3′. The density of mRNA bands was calculated by UN-SCAN-IT gel version 5.1 (Silk Scientific Inc. Orem, UT, USA).

### Transient transfection of expression vectors

HA-tagged wild type cyclin D1 (WT-cyclin D1) or HA-tagged T286A cyclin D1 (T286A-cyclin D1) construct was purchased from Addgene (Cambridge, MA, USA). WT- or T286A-cyclin D1 was transfected to SW480 cells for 48 h using the PolyJet DNA transfection reagent (SignaGen Laboratories, Ijamsville, MD, USA).

### Statistical analysis

All the data are shown as mean ± SD (standard deviation). Statistical analysis was performed with one-way ANOVA followed by Dunnett’s test. Differences with *P < 0.05 were considered statistically significant.

## Results

### STB and STL reduce the cell viability and cyclin D1 level in SW480 cells

**Fig. 1:**
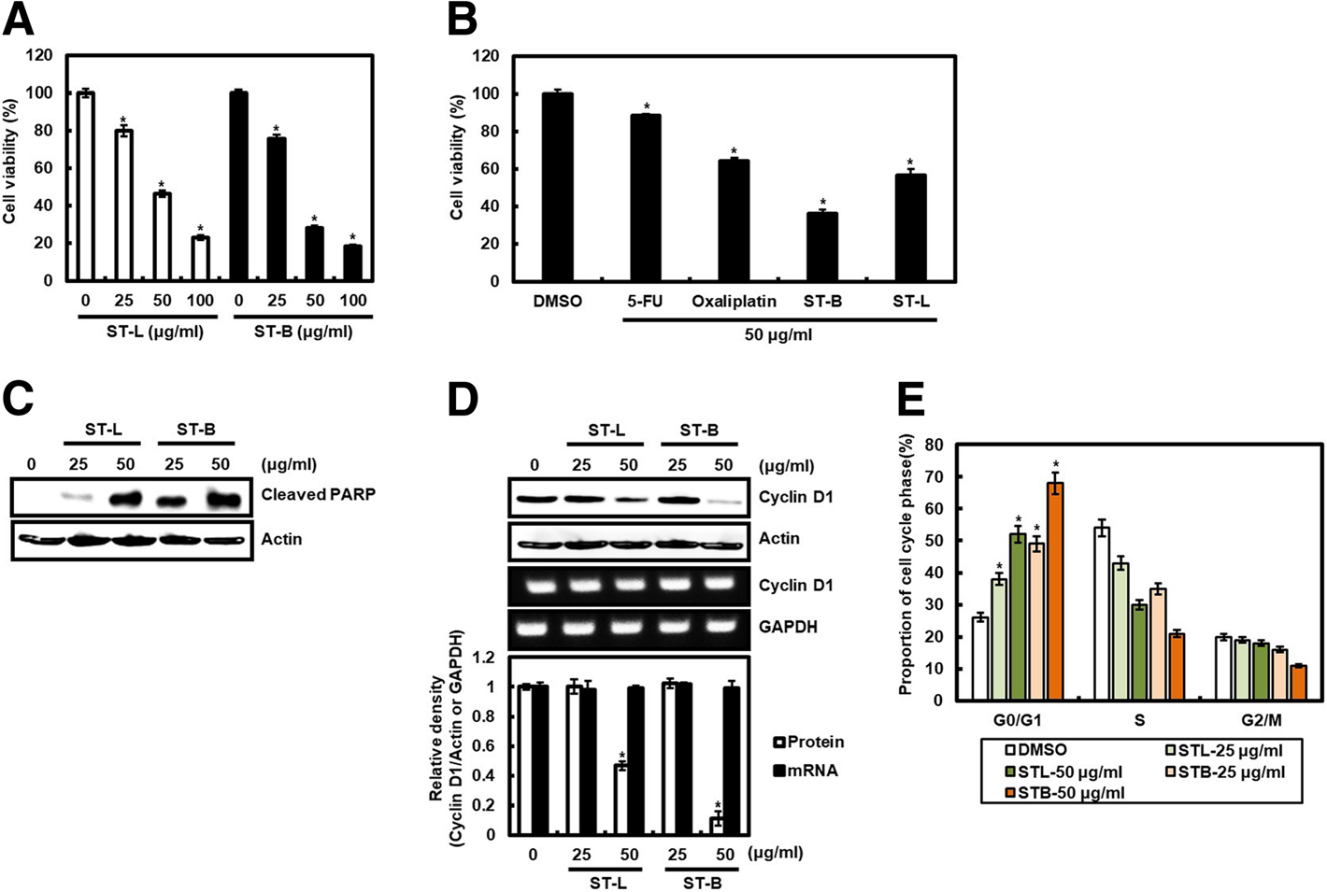
Effect of STL and STB on the cell viability, and the level of cleaved PARP and cyclin D1. **a** and **b** Cell viability of SW480 cells analyzed by MTT assay. ^*^P < 0.05 compared to the cells without (**c**) The cleaved PARP level analyzed by Western blot analysis in SW480 cells treated with STL and STB for 24 h. Actin was used as loading control. **d** The protein and mRNA level of cyclin D1 analyzed by Western blot analysis and RT-PCR in SW480 cells treated with STL and STB for 24 h. Actin or GAPDH was used as loading control. The density of protein and mRNA bands was calculated using the software UN-SCAN-IT gel version 5.1 (Silk Scientific Inc. Orem, UT, USA). ^*^P < 0.05 compared to the cells without STL and STB. **e** Proportions of the cycle phase analyzed by flow cytometry in in SW480 cells treated with STL and STB for 24 h. ^*^P < 0.05 compared to the cells without STL and STB.

To compare the inhibitory effect of STB and STL against the cell viability, STB or STL was treated to SW480 cells for 24 h, and then MTT assay was performed. STL and STB inhibited the viability of SW480 cells by 20.1 and 24.3% at 25 μg/ml, 53.6 and 72.2% at 50 μg/ml, and 77.0 and 82.0% at 100 μg/ml, respectively (Fig. 1a). IC_50_ of STL and STB was 48.51 ± 1.26 μg/ml and 31.93 ± 2.32 μg/ml, respectively. We compared the effect of STB or STL on the viability of SW480 cells with 5-FU and oxaliplatin as anticancer drugs used in the treatment of colorectal cancer. The inhibitory effect of 5-FU, oxaliplatin, STB or STL was 11.4, 35.6, 63.6% or 43.2% at 50 μg/ml, respectively (Fig. 1b). We evaluated the effect of STL and STB on the cleavage of PARP to investigate whether STL and STB affect apoptosis in SW480 cells. Treatment of STL and STB increased the cleavage of PARP in SW480 cells (Fig. 1c).

Because cyclin D1 not only induces over-proliferation of cancer cells, but also regulates apoptosis [11], and increased cyclin D1 protein level is observed in human colorectal cancer [35], the effect of STL and STB on cyclin D1 level was investigated. STL and STB decreased cyclin D1 protein level at 50 μg/ml, but not cyclin D1 mRNA level (Fig. 1d). To investigate that decrease of cyclin D1 protein level by STL and STB affects cell cycle arrest, the cell cycle distribution of SW480 cells treated with STL and STB were analyzed by flow cytometry. From the result (Fig. 1e), STL and STB induced the accumulation of G0/G1 phase in a dose-dependent manner.

### STB and STL induce cyclin D1 proteasomal degradation through inducing phosphorylation of cyclin D1 threonine-286

**Fig. 2:**
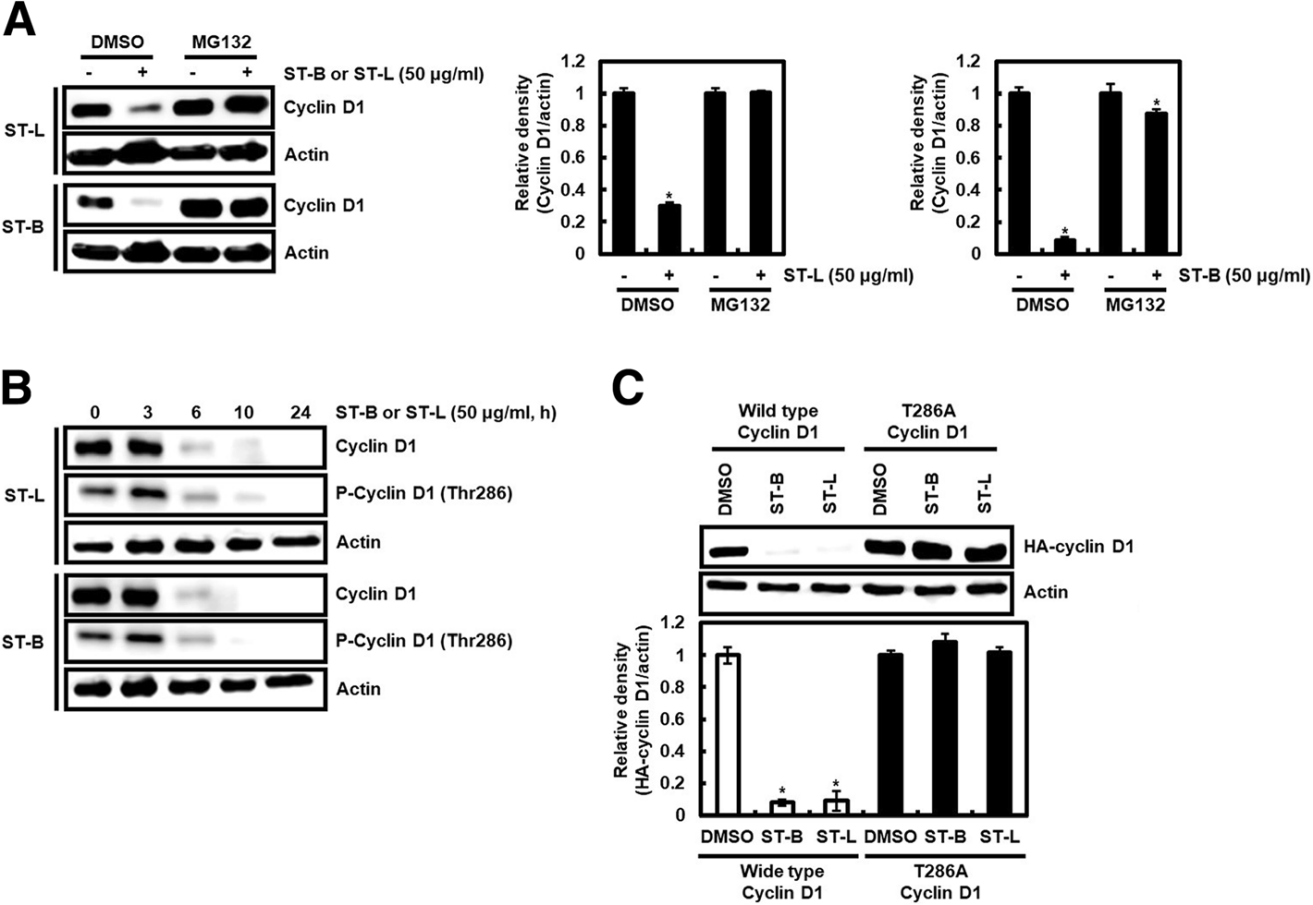
Induction of cyclin D1 degradation by STL and STB through Thr286 phosphorylation of cyclin D1. **a** SW480 cells were pretreated with MG132 (20 μM) and then co-treated with STL and STB for additional 6 h. **b** SW480 cells were treated with STL and STB for the indicated times. (**c**) SW480 cells were transfected with HA-tagged wild type cyclin D1 construct or HA-tagged T286A cyclin D1 construct for 48 h, and then co-treated with STL and STB for the additional 6 h. Actin was used as loading control. ^*^P < 0.05 compared to the cells without STL and STB

We observed that STL and STB attenuate cyclin D1 protein level, but not mRNA level, indicating that STL and STB may decrease cyclin D1 protein stability. To evaluate whether STL and STB induce cyclin D1 protein stability, STL or STB was treated to the cells in absence or presence of MG132 (proteasome inhibitor). Decreased cyclin D1 protein by STL and STB was observed in SW480 cells without MG132. However, pretreatment of MG132 attenuated decrease of cyclin D1 protein by STL and STB (Fig. 2a), which indicating that STL and STB may induce cyclin D1 degradation.

The threonine-286 (Thr286) phosphorylation of cyclin D1 is reported to contribute to cyclin D1 degradation [36]. Thus, we investigated effect of STL and STB on Thr286 phosphorylation of cyclin D1. Thr286 phosphorylation of cyclin D1 by STL and STB was observed to be occurred earlier than the decrease of cyclin D1 protein level by STL and STB (Fig. 2b). For confirming that cyclin D1 degradation by STL or STB results from Thr286 phosphorylation of cyclin D1, STB or ST was treated to SW480 cells transfected with WT-cyclin D1 or T286A-cyclin D1. HA-cyclin D1 was attenuated by STB or STL in the cells transfected with WT-cyclin D1, while the transfection of T286A-cyclin D1 blocked the reduction of HA-cyclin D1 level by STB or STL (Fig. 2c), which indicating that Thr286 phosphorylation of cyclin D1 may contribute to cyclin D1 degradation by STB or STL.

### Cyclin D1 proteasomal degradation by STB and STL is dependent on GSK3β-induced Thr286 phosphorylation of cyclin D1

**Fig. 3:**
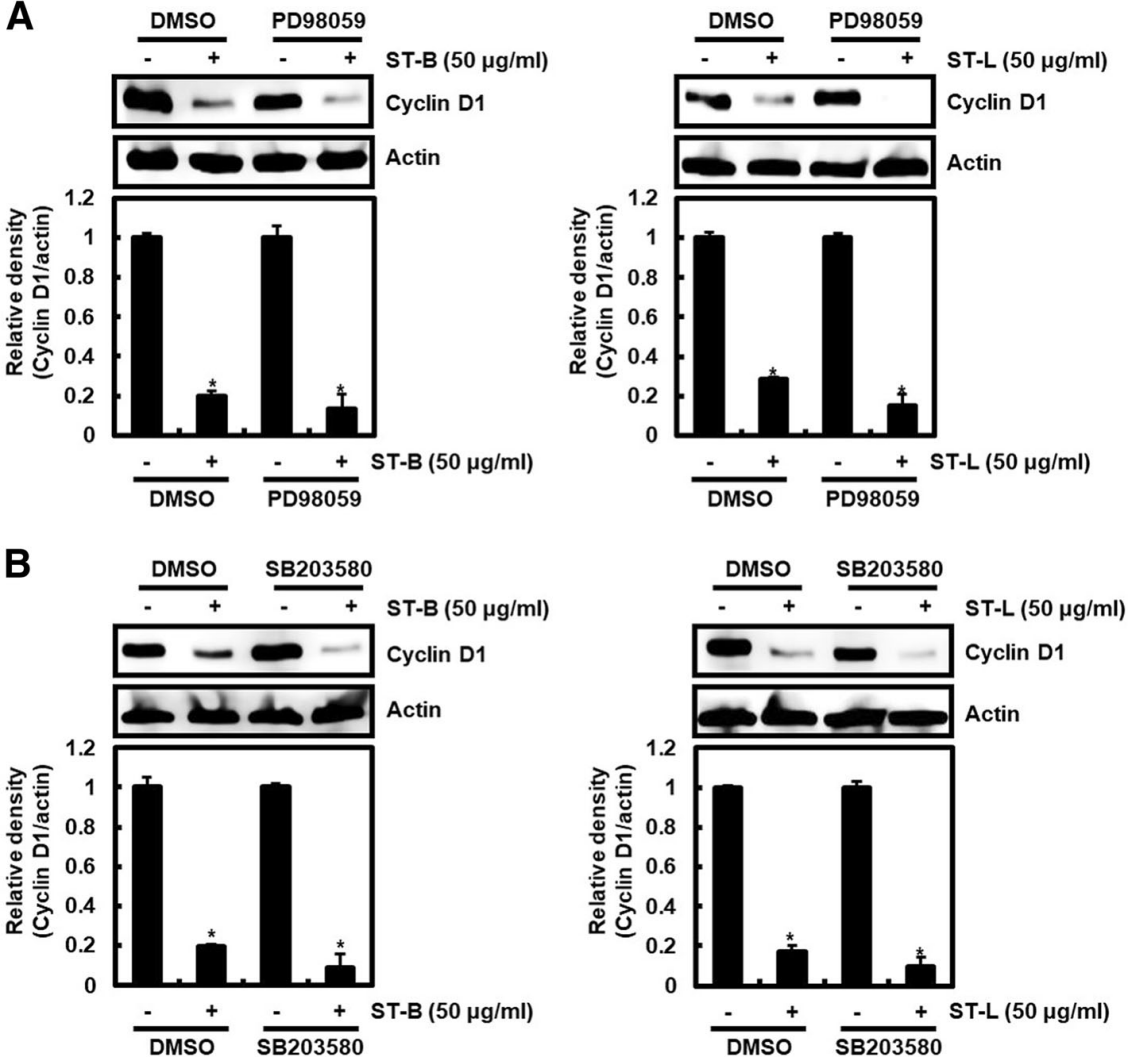
Cyclin D1 degradation by STL and STB is independent on ERK1/2 and p38. **a** and **b** SW480 cells were treated with PD98059 (20 μM) or SB203580 (20 μM) for 2 h, and then co-treated with STL and STB for the additional 6 h. Actin was used as loading control. ^*^P < 0.05 compared to the cells without STL and STB

**Fig. 4:**
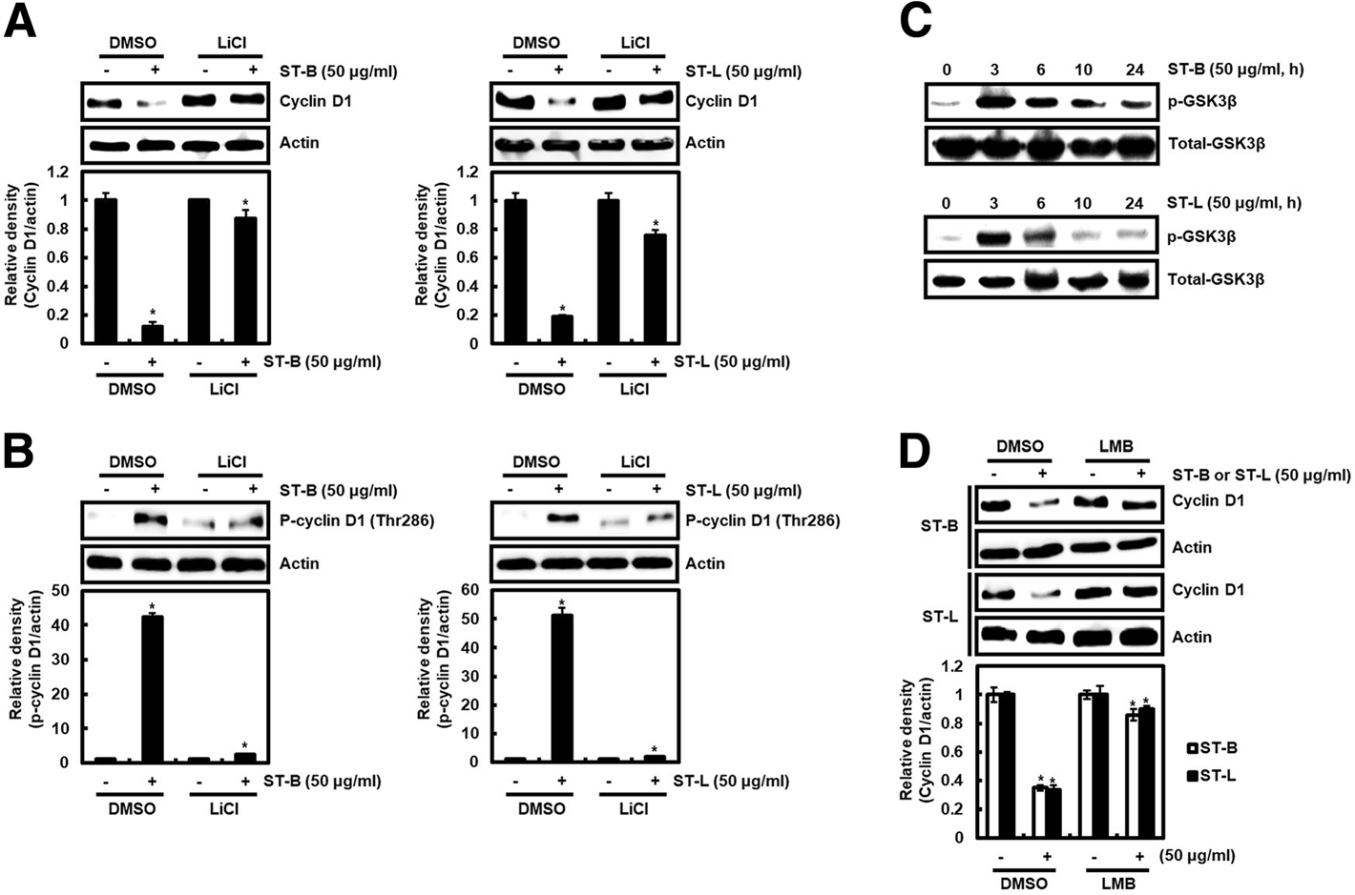
Cyclin D1 degradation by STL and STB is dependent on GSK3β. **a** SW480 cells were treated with LiCl (20 mM) for 2 h, and then co-treated with STL and STB for the additional 6 h. **b** SW480 cells were treated with LiCl (20 mM) for 2 h, and then co-treated with STL and STB for the additional 1 h. **c** SW480 cells were treated with STL and STB for the indicated times. **d** SW480 cells were treated with LMB (50 ng/ml) for 2 h, and then co-treated with STL and STB for the additional 6 h. Actin was used as loading control. ^*^P < 0.05 compared to the cells without STL and STB

Thr286 phosphorylation-dependent cyclin D1 degradation has been reported to be regulated by kinases such as extracellular signal-regulated kinase 1/2 (ERK1/2), p38, glycogen synthase kinase 3 beta (GSK3β) [37–40]. Thus, STB or STL was treated to SW480 cells in absence or presence of PD98059 (ERK1/2 inhibitor), SB203580 (p38 inhibitor) or LiCl (GSK3β inhibitor). Decreased cyclin D1 protein by STB and STL was observed in SW480 cells in absence or presence of PD98059 or SB203580 (Fig. 3a and b), which indicating cyclin D1 degradation by STB and STL may be independent on ERK1/2 or p38. However, we observed that pretreatment of LiCl suppressed the decrease of cyclin D1 protein by STB and STL (Fig. 4a). Furthermore, LiCl inhibited Thr286 STB and STL-mediated phosphorylation of cyclin D1 (Fig. 4b). These data mean that cyclin D1 degradation by STB and STL may be dependent on GSK3β-dependent Thr286 phosphorylation of cyclin D1. To investigate whether STB and STL activate GSK3β, the phosphorylation of GSK3β as an active form by STB or STL was detected. STB and STL increased the phosphorylation of GSK3β (Fig. 4c).

GSK3β-dependent Thr286 phosphorylation of cyclin D1 induces the redistribution of cyclin D1 from cytoplasm to the nucleus, which is known to be involved in the degradation of cyclin D1 [36]. Thus, we investigated that the inhibition of cyclin D1 redistribution from cytoplasm to the nucleus by LMB as an inhibitor of nuclear export affects cyclin D1 degradation mediated by STB and STL. LMB attenuated cyclin D1 degradation by STB and STL compared to the treatment of STB and STL in absence of LMB (Fig. 4d), which indicating that STB and STL may induce cyclin D1 degradation through GSK3β-dependent Thr286 phosphorylation of cyclin D1 and subsequent cyclin D1 redistribution from cytoplasm to the nucleus.

### STB or STL upregulates HO-1 expression

**Fig. 5:**
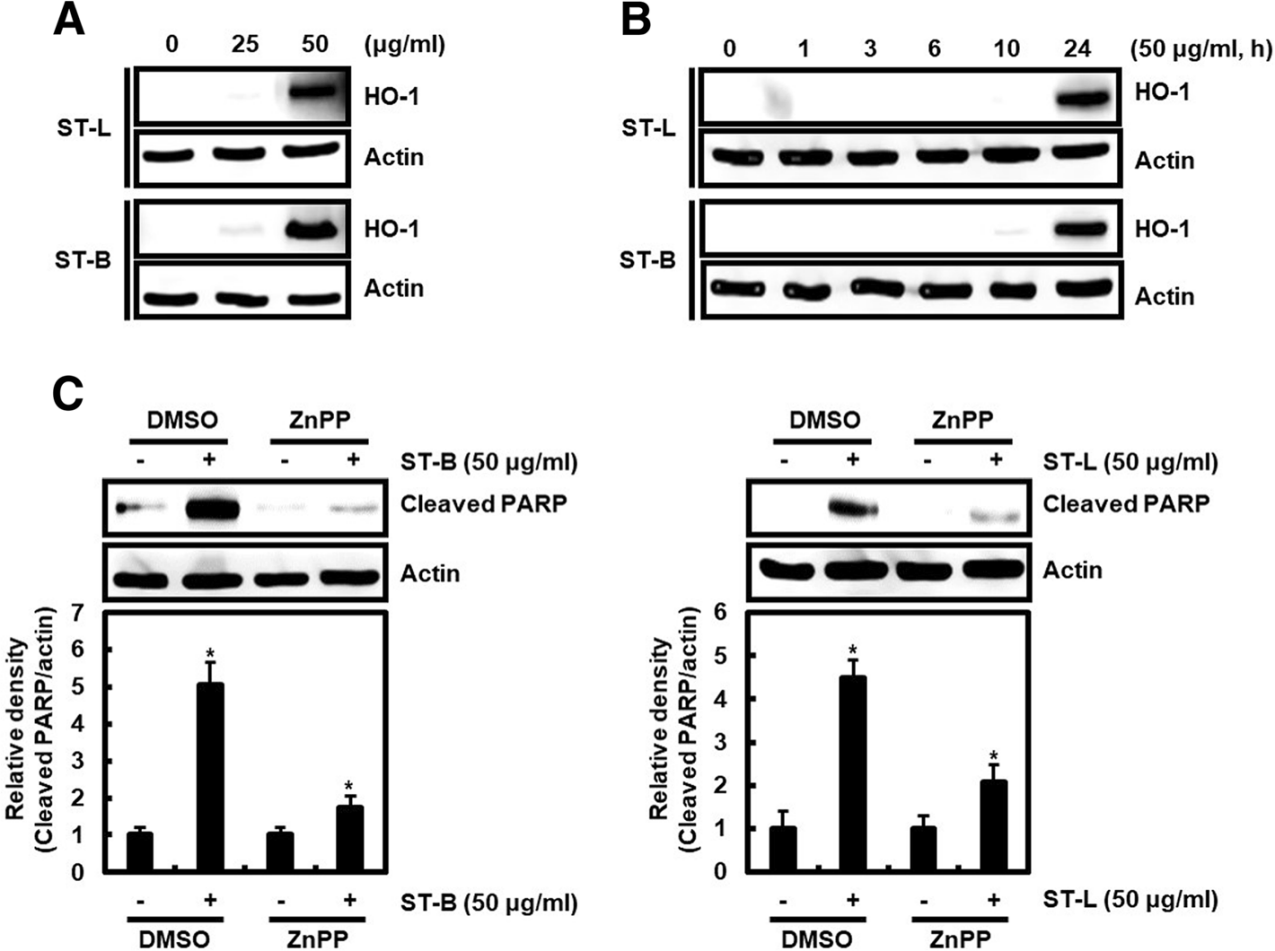
STL and STB increase HO-1 expression. **a** SW480 cells were treated with STL and STB for 24 h. **b** SW480 cells were treated with STL and STB for the indicated times. **c** SW480 cells were treated with ZnPP (1 μM) for 2 h and then co-treated with STL and STB for the additional 24 h. Actin was used as loading control. ^*^P < 0.05 compared to the cells without STL and STB

Heme oxygenase-1 (HO-1) exerts anti-tumor activity in prostate and colorectal cancers [26, 41, 42]. HO-1 activation reduced the viability of human colorectal cancer cells [43]. Thus, we evaluated whether STB and STL affects HO-1 expression in SW480 cells. In Fig. 5a, 25 μg/ml of STL and STB slightly increased HO-1 expression, but HO-1 expression was dramatically increased at 50 μg/ml of STL and STB. We observed that the significant increase of HO-1 expression occurred at 24 h after the treatment of STL and STB (Fig. 5b). To determine that HO-1 expression by STB and STL contributes to apoptosis, STB or STL was treated to SW480 cells in absence or presence of ZnPP (HO-1 inhibitor). PARP was cleaved by the treatment of STB and STL in absence of ZnPP, but the presence of ZnPP inhibited the cleavage of PARP by STB and STL (Fig. 5c). These results indicate that HO-1 may be contribute to apoptosis by STB and STL.

### Nrf2 activation dependent on ROS-induced p38 activation contributes to the elevated HO-1 expression by STB and STL

**Fig. 6:**
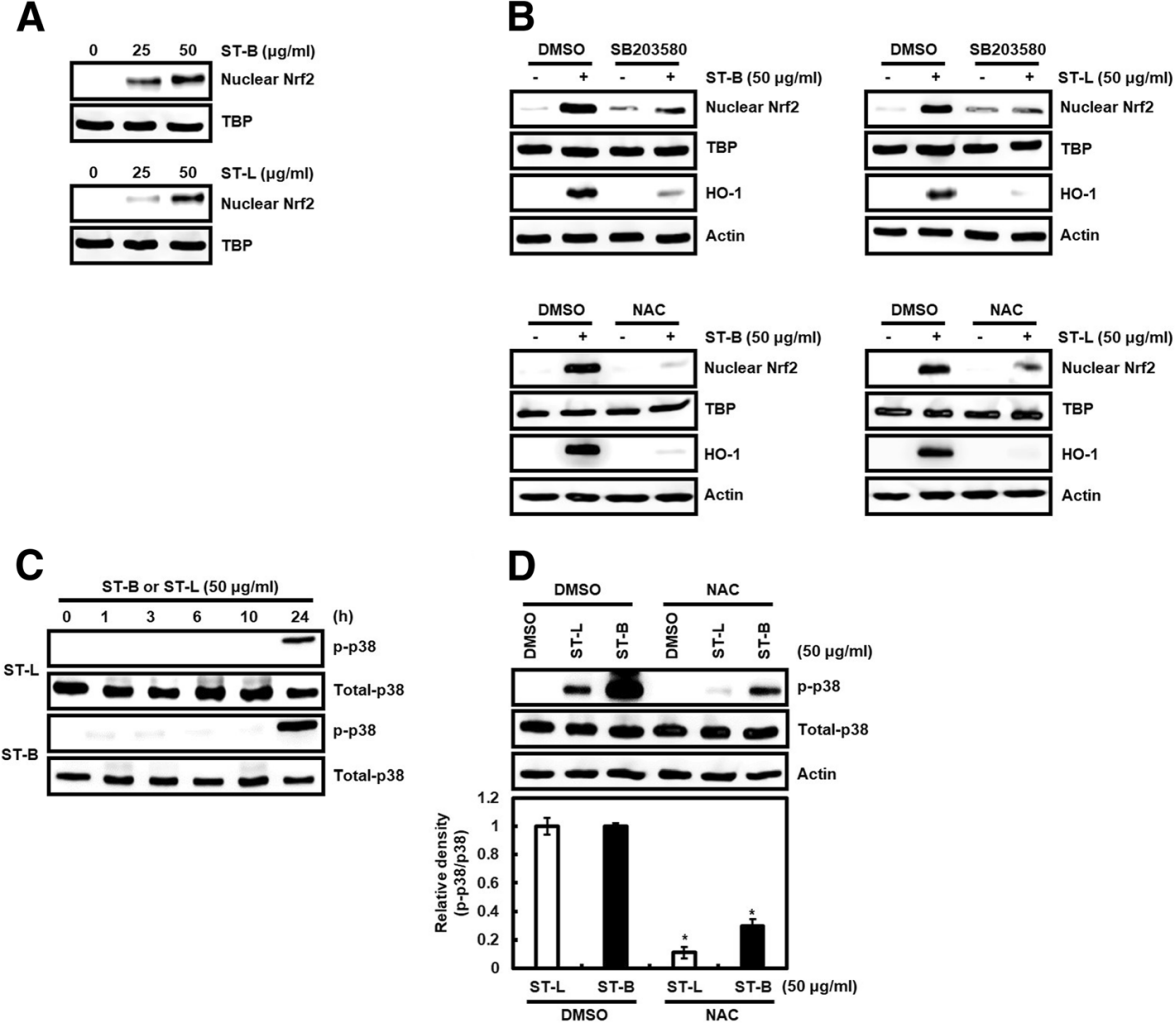
HO-1 expression by STL and STB is through Nrf2 activation dependent on ROS-mediated p38 activation. **a** SW480 cells were treated with STL and STB for 24 h. **b** SW480 cells were pretreated with SB203580 (20 μM) or NAC (20 mM) for 2 h, and then co-treated with STL and STB for the additional 24 h. **c** SW480 cells were treated with STL and STB for the indicated times. **d** SW480 cells were treated with NAC (20 mM) for 2 h, and then co-treated with STL and STB for the additional 24 h. Actin, TBP or total-p38 was used as loading control. ^*^P < 0.05 compared to the cells without STL and STB

Under reactive oxygen species (ROS)-mediated p38 activation, nuclear factor erythroid 2-related factor-2 (Nrf2) was translocated into the nucleus and binds to antioxidant response element (ARE) of HO-1 promoter region, which contributes to HO-1 expression [44, 45]. Firstly, we evaluated whether STB and STL induces the nuclear accumulation of Nrf2 in SW480 cells. As shown in Fig. 6a, the nuclear level of Nrf2 was increased by the treatment of STB and STL (Fig. 6a). Thus, we also investigated whether p38 and ROS affect the nuclear accumulation of Nrf2 induced by STB and STL in SW480 cells. p38 inhibition by SB203580 blocked the nuclear accumulation of Nrf2 induced by ST-B and ST-L, which resulted to the attenuation of HO-1 expression (Fig. 6b). Blocking Nrf2 nuclear accumulation and HO-1 expression mediated by STB and STL was also observed in SW480 cells treated with NAC as a ROS scavenger (Fig. 6b). Because p38 activation has been reported to be the downstream of ROS, we investigated whether ROS affects p38 activation by STB and STL. As shown in Fig. 6c, STB and STL induced p38 phosphorylation in absence of NAC, but NAC treatment attenuated p38 phosphorylation by STB and STL. These data indicate that STB and STL may induce apoptosis through Nrf2-dependent HO-1 expression via ROS-induced p38 activation.

## Discussion

In this study, we observed that STL and STB reduce the cell viability and induce apoptosis in SW480 cells. STL and STB decreased cyclin D1 protein level but not mRNA level, which indicates that STL and STB may affect cyclin D protein stability. Indeed, cyclin D1 protein has been reported to be upregulated by the gene amplification or defective proteasomal degradation [7, 46]. Furthermore, there is a growing evidence that has been reported that elevated cyclin D1 protein in cancers is a consequence of the defective proteasomal degradation pathway of cyclin D1 protein [47]. Thus, cyclin D1 degradation is considered as a promising target for anticancer drugs [36]. Cyclin D1 degradation by STL and STB was mitigated in SW480 cells treated with MG132 as a proteasome inhibitor. These results indicate that STL and STB-induced cyclin D1 degradation may contribute to the downregulation of cyclin D1 protein level in SW480 cells.

Cyclin D1 is degraded after Thr286 phosphorylation induced by activated ERK1/2, p38 and GSK3β [36–40]. Cyclin D1 has been reported to exhibit resistance to its degradation in the cyclin D1 mutant T286A [37]. In this study, we observed that Thr286 phosphorylation of cyclin D1 occurs faster than degradation of cyclin D1 in SW480 cells treated with STL and STB. Furthermore, when Thr286 was converted to alanine, cyclin D1 degradation by STL and STB did not occur in SW480 cells. These results indicate that Thr286 phosphorylation of cyclin D1 may be an essential step in inducing cyclin D1 degradation by STL and STB. In the determination of the upstream kinases such as ERK1/2, p38 and GSK3β, cyclin D1 degradation was observed in SW480 cells treated with ERK1/2 and p38 inhibitor, which indicates that STL and STB-induced degradation of cyclin D1 is independent on ERK1/2 and p38. However, the inhibition of GSK3β mitigated Thr286 phosphorylation and degradation of cyclin D1 by STL and STB. These results suggest that cyclin D1 degradation followed by Thr286 phosphorylation of cyclin D1 may result from GSK3β activation. Actually, STL and STB were observed to activate GSK3β. Indeed, many phytochemicals with anti-cancer activity induced GSK3β-dependent cyclin D1 degradation [48–50]. Furthermore, our results showed that LMB treatment for the inhibition of nuclear-to-cytoplasmic redistribution of cyclin D1 attenuated STL and STB-mediated degradation of cyclin D1. Although GSK3β is a cytoplasmic protein, activated GSK3β translocates into the nucleus and phosphorylates Thr286 of cyclin D1, which contributes to nuclear-to-cytoplasmic redistribution of cyclin D1 and subsequent degradation of cyclin D1 [36, 37].

Some phytochemicals such as curcumin and sulforaphane exerts anti-tumorigenic activity through HO-1 induction [51, 52]. Our results showed that STL and STB increased HO-1 expression, and inhibition of HO-1 attenuated PARP cleavage in SW480 cells, which indicates that HO-1 may be a potential molecular target for the induction of apoptosis by STL and STB. It has been reported that ROS-mediated p38 activation enhances the translocation of Nrf2 into the nucleus and nuclear Nrf2 binds to ARE of HO-1 promoter region, which contributes to HO-1 expression [44, 45]. In this study, STL and STB induced ROS-dependent p38 activation. In addition, STL and STB increased nuclear Nrf2 protein level dependent on ROS and p38, which resulted in HO-1 expression. These results suggest that STL and STB may increase HO-1 expression through activating Nrf2 via ROS-dependent p38 activation.

*S. thea* has been reported to have various bioactive compounds such as taraxerol, quercetin, syringic acid, myricetrin, kaempferol and daucosterol [53–55]. There is a growing evidence that these compounds anti-cancer activity [56–60]. However, in order to standardize STL and STB for the industrialization, it is necessary to analyze the representative compounds related to anti-cancer activity of STL and STB.

## Conclusion

**Fig. 7:**
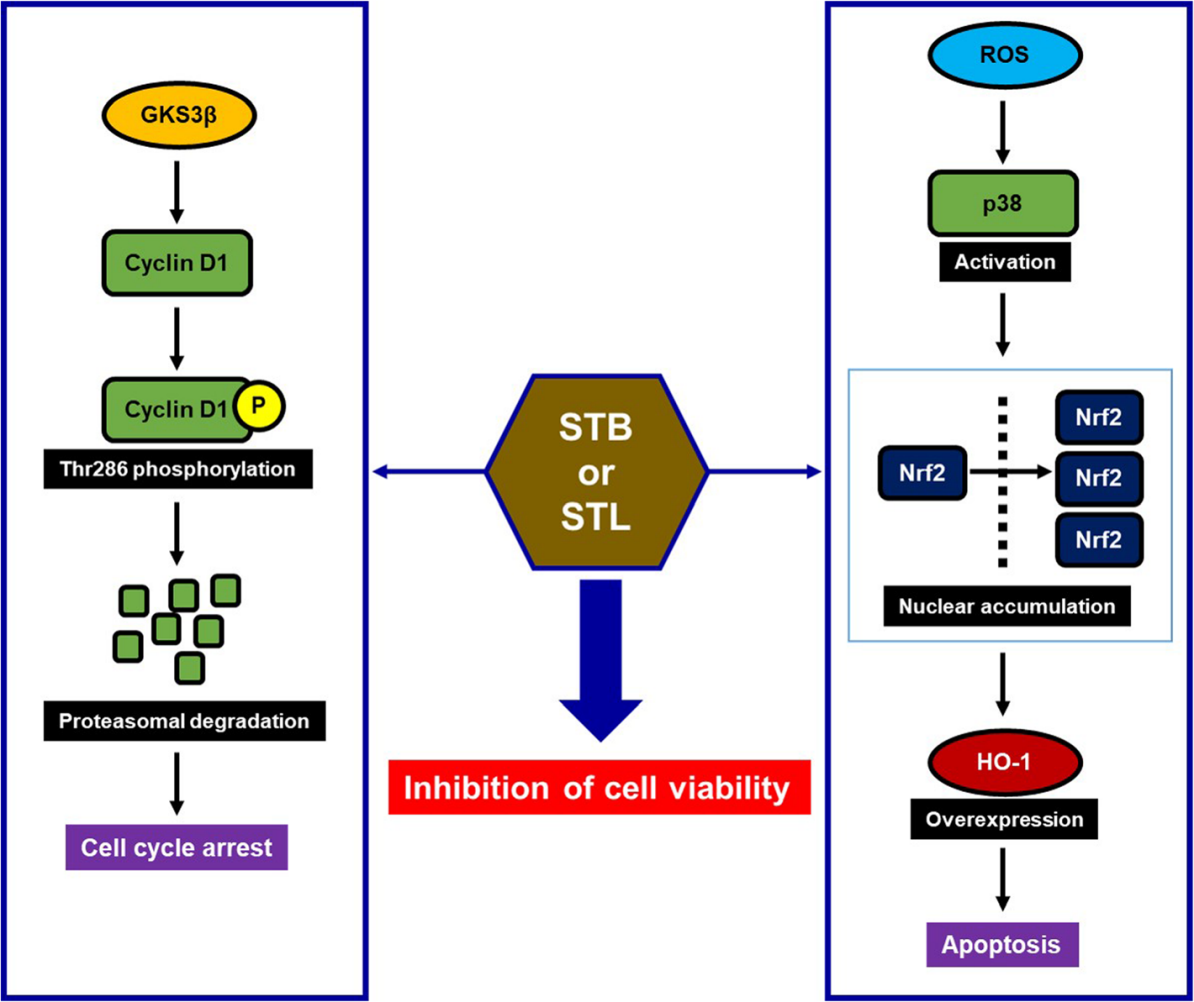
The proposed cascade of events for STL and STB-induced reduction of cell viability in human colorectal cancer cells

In conclusion, the current study demonstrated that STL and STB induced cyclin D1 degradation through GSK3β-dependent phosphorylation of cyclin D1 threonine-286, and increased HO-1 expression through activating Nrf2 via ROS-dependent p38 activation, which resulted in the decrease of the viability in SW480 cells (Fig. 7). These findings suggest that STL and STB may have great potential for the development of anti-cancer drug for human colorectal cancer. However, the anti-cancer effect of STL and STB in vivo and the identification of major compound from STL and STB with anti-cancer effect need further studies.

## Abbreviations

5-FU: 5-Fluorouracil
ARE: Antioxidant response element
ERK1/2: Extracellular signal-regulated kinase 1/2
GSK3β: Glycogen synthase kinase 3 beta
HO-1: Heme oxygenase-1
LMB: Leptomycin B
MTT: 3-(4,5-dimethylthizaol-2-yl)-2,5-diphenyl tetrazolium bromide
Nrf2: Nuclear factor erythroid 2-related factor-2
PARP: Poly (ADP-ribose) polymerase
ROS: Reactive oxygen species
RT-PCR: Reverse transcriptase-polymerase chain reaction
STB: Branch extracts of *Sageretia thea*
STL: Leave extracts of *Sageretia thea*
Thr286: Threonine-286
ZnPP: Zinc protoporphyrin IX

## References

[1] Siegel RL, Miller KD, Jemal A (2018). Cancer statistics, 2018. CA Cancer J Clin.

[2] Arnold M, Sierra MS, Laversanne M, Soerjomataram I, Jemal A, Bray F (2017). Global patterns and trends in colorectal cancer incidence and mortality. Gut.

[3] Meyerhardt JA, Mayer RJ (2005). Systemic therapy for colorectal cancer. N Engl J Med.

[4] Polachi N, Subramaniyan B, Nagaraja P, Rangiah K, Ganeshan M (2018). Extract from *Butea monosperma* inhibits β-catenin/Tcf signaling in SW480 human colon cancer cells. Gene Rep.

[5] Benarba B, Belabid L, Righi K, Bekkar AA, Elouissi M, Khaldi A, Hamimed A (2015). Study of medicinal plants used by traditional healers in Mascara (north west of Algeria). J Ethnopharmacol.

[6] Shukla S, Mehta A (2015). Anticancer potential of medicinal plants and their phytochemicals: a review. Braz J Bot.

[7] Musgrove EA, Caldon CE, Barraclough J, Stone A, Sutherland RL (2011). Cyclin D as a therapeutic target in cancer. Nat Rev Cancer.

[8] Coqueret O (2002). Linking cyclins to transcriptional control. Gene.

[9] Yasui M, Yamamoto H, Ngan CY, Damdinsuren B, Sugita Y, Fukunaga H, Gu J, Maeda M, Takemasa I, Ikeda M, Fujio Y, Sekimoto M, Matsuura N, Weinstein IB, Monden M (2006). Antisense to cyclin D1 inhibits vascular endothelial growth factor-stimulated growth of vascular endothelial cells: implication of tumor vascularization. Clin Cancer Rese.

[10] Zhang L, Fried FB, Guo H, Friedman AD (2008). Cyclin-dependent kinase phosphorylation of RUNX1/AML1 on 3 sites increases transactivation potency and stimulates cell proliferation. Blood.

[11] Roue G, Pichereau V, Lincet H, Colomer D, Sola B (2008). Cyclin D1 mediates resistance to apoptosis through upregulation of molecular chaperones and consequent redistribution of cell death regulators. Oncogene.

[12] Driscoll B, Buckley S, Barsky L, Weinberg K, Anderson KD, Warburton D (1999). Abrogation of cyclin D1 expression predisposes lung cancer cells to serum deprivation-induced apoptosis. Am J Phys.

[13] Sauter ER, Nesbit M, Litwin S, Klein-Szanto AJ, Cheffetz S, Herlyn M (1999). Antisense cyclin D1 induces apoptosis and tumor shrinkage in human squamous carcinomas. Cancer Res.

[14] Albanese C, D'Amico M, Reutens AT, Fu M, Watanabe G, Lee RJ, Kitsis RN, Henglein B, Avantaggiati M, Somasundaram K, Thimmapaya B, Pestell RG (1999). Activation of the cyclin D1 gene by the E1A-associated protein p300 through AP-1 inhibits cellular apoptosis. J Biol Chem.

[15] Bahnassy AA, Zekri AR, El-Houssini S, El-Shehaby AM, Mahmoud MR, Abdallah S, El-Serafi M (2004). Cyclin a and cyclin D1 as significant prognostic markers in colorectal cancer patients. BMC Gastroenterol.

[16] Balcerczak E, Pasz-Walczak G, Kumor P, Panczyk M, Kordek R, Wierzbicki R, Mirowski M (2005). Cyclin D1 protein and CCND1 gene expression in colorectal cancer. Eur J Surg Oncol.

[17] Podkalicka P, Mucha O, Jozkowicz A, Dulak J, Loboda A. Heme oxygenase inhibition in cancers: possible tools and targets. Contemp. Oncol. (Pozn) 2018;22(1A):23–32. 10.5114/wo.2018.73879 PMC5885082 29628790

[18] Castilho A, Aveleira CA, Leal EC, Simoes NF, Fernandes CR, Meirinhos RI, Baptista FI, Ambrosio AF (2012). Heme oxygenase-1 protects retinal endothelial cells against high glucose- and oxidative/nitrosative stress-induced toxicity. PLoS One.

[19] Chen YC, Chow JM, Lin CW, Wu CY, Shen SC (2006). Baicalein inhibition of oxidative-stress-induced apoptosis via modulation of ERKs activation and induction of HO-1 gene expression in rat glioma cells C6. Toxicol Appl Pharmacol.

[20] Lee IT, Luo SF, Lee CW, Wang SW, Lin CC, Chang CC, Chen YL, Chau LY, Yang CM (2009). Overexpression of HO-1 protects against TNF-alpha-mediated airway inflammation by down-regulation of TNFR1-dependent oxidative stress. Am J Pathol.

[21] Lin HY, Shen SC, Lin CW, Yang LY, Chen YC (2007). Baicalein inhibition of hydrogen peroxide-induced apoptosis via ROS-dependent heme oxygenase 1 gene expression. Biochim Biophys Acta.

[22] Berberat PO, Dambrauskas Z, Gulbinas A, Giese T, Giese N, Kunzli B, Autschbach F, Meuer S, Buchler MW, Friess H (2005). Inhibition of heme oxygenase-1 increases responsiveness of pancreatic cancer cells to anticancer treatment. Clin Cancer Res.

[23] Was H, Cichon T, Smolarczyk R, Rudnicka D, Stopa M, Chevalier C, Leger JJ, Lackowska B, Grochot A, Bojkowska K, Ratajska A, Kieda C, Szala S, Dulak J, Jozkowicz A (2006). Overexpression of heme oxygenase-1 in murine melanoma: increased proliferation and viability of tumor cells, decreased survival of mice. Am J Pathol.

[24] Ciesla M, Marona P, Kozakowska M, Jez M, Seczynska M, Loboda A, Bukowska-Strakova K, Szade A, Walawender M, Kusior M, Stepniewski J, Szade K, Krist B, Yagensky O, Urbanik A, Kazanowska B, Dulak J, Jozkowicz A (2016). Heme Oxygenase-1 controls an HDAC4-miR-206 pathway of oxidative stress in rhabdomyosarcoma. Cancer Res.

[25] Hill M, Pereira V, Chauveau C, Zagani R, Remy S, Tesson L, Mazal D, Ubillos L, Brion R, Asghar K, Mashreghi MF, Kotsch K, Moffett J, Doebis C, Seifert M, Boczkowski J, Osinaga E, Anegon I (2005). Heme oxygenase-1 inhibits rat and human breast cancer cell proliferation: mutual cross inhibition with indoleamine 2,3-dioxygenase. FASEB J.

[26] Gueron G, De Siervi A, Ferrando M, Salierno M, De Luca P, Elguero B, Meiss R, Navone N, Vazquez ES (2009). Critical role of endogenous heme oxygenase 1 as a tuner of the invasive potential of prostate cancer cells. Mol Cancer Res.

[27] Yin H, Fang J, Liao L, Maeda H, Su Q (2014). Upregulation of heme oxygenase-1 in colorectal cancer patients with increased circulation carbon monoxide levels, potentially affects chemotherapeutic sensitivity. BMC Cancer.

[28] Zou C, Zhang H, Li Q, Xiao H, Yu L, Ke S, Zhou L, Liu W, Wang W, Huang H, Ma N, Liu Q, Wang X, Zhao W, Zhou H, Gao X (2011). Heme oxygenase-1: a molecular brake on hepatocellular carcinoma cell migration. Carcinogenesis.

[29] Chung SK, Chen CY, Blumberg JB (2009). Flavonoid-rich fraction from *Sageretia theezans* leaves scavenges reactive oxygen radical species and increases the resistance of low-density lipoprotein to oxidation. J Med Food.

[30] Hyun TK, Song SC, Song CK, Kim JS (2015). Nutritional and nutraceutical characteristics of *Sageretia theezans* fruit. J Food Drug Anal.

[31] Ko GA, Shrestha S, Kim CS (2018). *Sageretia thea* fruit extracts rich in methyl linoleate and methyl linolenate downregulate melanogenesis via the Akt/GSK3beta signaling pathway. Nutr Res Pract.

[32] Park JC, Hur JM, Park JG, Hatano T, Yoshida T, Miyashiro H, Min BS, Hattori M (2002). Inhibitory effects of Korean medicinal plants and camelliatannin H from *Camellia japonica* on human immunodeficiency virus type 1 protease. Phytother Res.

[33] Ko GA, Son M, Cho SK (2016). Comparative evaluation of free radical scavenging activities and cytotoxicity of various solvent fractions of Sandong *Sageretia thea* (Osbeck) M.C. Johnst. Branches. Food Sci. Biotechnol.

[34] Balaji C, Muthukumaran J, Vinothkumar R, Nalini N (2014). Anticancer effects of sinapic acid on human colon cancer cell lines HT-29 and SW480. Int J Pharm Biol Arch.

[35] Li Y, Wei J, Xu C, Zhao Z, You T (2014). Prognostic significance of cyclin D1 expression in colorectal cancer: a meta-analysis of observational studies. PLoS One.

[36] Alao JP (2007). The regulation of cyclin D1 degradation: roles in cancer development and the potential for therapeutic invention. Mol Cancer.

[37] Okabe H, Lee SH, Phuchareon J, Albertson DG, McCormick F, Tetsu O (2006). A critical role for FBXW8 and MAPK in cyclin D1 degradation and cancer cell proliferation. PLoS One.

[38] Casanovas O, Miro F, Estanyol JM, Itarte E, Agell N, Bachs O (2000). Osmotic stress regulates the stability of cyclin D1 in a p38SAPK2-dependent manner. J Biol Chem.

[39] Thoms HC, Dunlop MG, Stark LA (2007). p38-mediated inactivation of cyclin D1/cyclin-dependent kinase 4 stimulates nucleolar translocation of RelA and apoptosis in colorectal cancer cells. Cancer Res.

[40] Diehl JA, Cheng M, Roussel MF, Sherr CJ (1998). Glycogen synthase kinase-3beta regulates cyclin D1 proteolysis and subcellular localization. Genes Dev.

[41] Becker JC, Fukui H, Imai Y, Sekikawa A, Kimura T, Yamagishi H, Yoshitake N, Pohle T, Domschke W, Fujimori T (2007). Colonic expression of heme oxygenase-1 is associated with a better long-term survival in patients with colorectal cancer. Scand J Gastroenterol.

[42] Kang KA, Maeng YH, Zhang R, Yang YR, Piao MJ, Kim KC, Kim GY, Kim YR, Koh YS, Kang HK, Hyun CL, Chang WY, Hyun JW (2012). Involvement of heme oxygenase-1 in Korean colon cancer. Tumour Biol.

[43] Andrés NC, Fermento ME, Gandini NA, Romero AL, Ferro A, Donna LG, Curino AC, Facchinetti MM (2014). Heme oxygenase-1 has antitumoral effects in colorectal cancer: involvement of p53. Exp Mol Pathol.

[44] Park EJ, Lim JH, Nam SI, Park JW, Kwon TK (2010). Rottlerin induces heme oxygenase-1 (HO-1) up-regulation through reactive oxygen species (ROS) dependent and PKC delta-independent pathway in human colon cancer HT29 cells. Biochimie.

[45] Nicco C, Batteux F (2017). ROS modulator molecules with therapeutic potential in cancers treatments. Molecules.

[46] Gillett C, Fantl V, Smith R, Fisher C, Bartek J, Dickson C, Barnes D, Peters G (1994). Amplification and overexpression of cyclin D1 in breast cancer detected by immunohistochemical staining. Cancer Res.

[47] Barbash O, Diehl JA (2008). SCF(Fbx4/alphaB-crystallin) E3 ligase: when one is not enough. Cell Cycle.

[48] Yu XJ, Han QB, Wen ZS, Ma L, Gao J, Zhou GB (2012). Gambogenic acid induces G1 arrest via GSK3beta-dependent cyclin D1 degradation and triggers autophagy in lung cancer cells. Cancer Lett.

[49] Cheng YH, Li LA, Lin P, Cheng LC, Hung CH, Chang NW, Lin C (2012). Baicalein induces G1 arrest in oral cancer cells by enhancing the degradation of cyclin D1 and activating AhR to decrease Rb phosphorylation. Toxicol Appl Pharmacol.

[50] Park GH, Song HM, Jeong JB (2016). The coffee diterpene kahweol suppresses the cell proliferation by inducing cyclin D1 proteasomal degradation via ERK1/2, JNK and GKS3beta-dependent threonine-286 phosphorylation in human colorectal cancer cells. Food Chem Toxicol.

[51] Cornblatt BS, Ye L, Dinkova-Kostova AT, Erb M, Fahey JW, Singh NK, Chen MS, Stierer T, Garrett-Mayer E, Argani P, Davidson NE, Talalay P, Kensler TW, Visvanathan K (2007). Preclinical and clinical evaluation of sulforaphane for chemoprevention in the breast. Carcinogenesis.

[52] Keum YS, Yu S, Chang PP, Yuan X, Kim JH, Xu C, Han J, Agarwal A, Kong AN (2006). Mechanism of action of sulforaphane: inhibition of p38 mitogen-activated protein kinase isoforms contributing to the induction of antioxidant response element-mediated heme oxygenase-1 in human hepatoma HepG2 cells. Cancer Res.

[53] Xu L, Yang X, Li B (1994). Chemical constituents of *Sageretia theezans* Brongn. Zhongguo Zhong yao za zhi.

[54] Chung SK, Kim YC, Takaya Y, Terashima K, Niwa M (2004). Novel flavonol glycoside, 7-O-methyl mearnsitrin, from *Sageretia theezans* and its antioxidant effect. J Agric Food Chem.

[55] Shen CJ, Chen CK, Lee SS (2009). Polar constituents from *Sageretia thea* leaf characterized by HPLC-SPE-NMR assisted approaches. J Chin Inst Chem.

[56] Hong JF, Song YF, Liu Z, Zheng ZC, Chen HJ, Wang SS (2016). Anticancer activity of taraxerol acetate in human glioblastoma cells and a mouse xenograft model via induction of autophagy and apoptotic cell death, cell cycle arrest and inhibition of cell migration. Mol Med Rep.

[57] Rauf A, Imran M, Khan IA, Ur-Rehman M, Gilani SA, Mehmood Z, Mubarak MS (2018). Anticancer potential of quercetin: a comprehensive review. Phytother Res.

[58] Abaza MS, Al-Attiyah R, Bhardwaj R, Abbadi G, Koyippally M, Afzal M (2013). Syringic acid from Tamarix aucheriana possesses antimitogenic and chemo-sensitizing activities in human colorectal cancer cells. Pharm Biol.

[59] Kim SH, Choi KC (2013). Anti-cancer effect and underlying mechanism(s) of kaempferol, a phytoestrogen, on the regulation of apoptosis in diverse cancer cell models. Toxicol Res.

[60] Zhao C, She T, Wang L, Su Y, Qu L, Gao Y, Xu S, Cai S, Shou C (2015). Daucosterol inhibits cancer cell proliferation by inducing autophagy through reactive oxygen species-dependent manner. Life Sci.

